# Differentiation grade as a risk factor for lymph node metastasis in T1 colorectal cancer

**DOI:** 10.1002/deo2.324

**Published:** 2023-12-28

**Authors:** Osamu Shiina, Shin‐ei Kudo, Katsuro Ichimasa, Yuki Takashina, Yuta Kouyama, Kenichi Mochizuki, Yuriko Morita, Takanori Kuroki, Shun Kato, Hiroki Nakamura, Shingo Matsudaira, Masashi Misawa, Noriyuki Ogata, Takemasa Hayashi, Kunihiko Wakamura, Naruhiko Sawada, Toshiyuki Baba, Tetsuo Nemoto, Fumio Ishida, Hideyuki Miyachi

**Affiliations:** ^1^ Digestive Disease Center Showa University Northern Yokohama Hospital Kanagawa Japan; ^2^ Department of Medicine National University of Singapore Singapore Singapore; ^3^ Department of Diagnostic Pathology Showa University Northern Yokohama Hospital Kanagawa Japan

**Keywords:** colorectal neoplasms, endoscopic mucosal resection, lymph nodes, neoplasm grading, risk factor

## Abstract

**Objectives:**

Japanese guidelines include high‐grade (poorly differentiated) tumors as a risk factor for lymph node metastasis (LNM) in T1 colorectal cancer (CRC). However, whether the grading is based on the least or most predominant component when the lesion consists of two or more levels of differentiation varies among institutions. This study aimed to investigate which method is optimal for assessing the risk of LNM in T1 CRC.

**Methods:**

We retrospectively evaluated 971 consecutive patients with T1 CRC who underwent initial or additional surgical resection from 2001 to 2021 at our institution. Tumor grading was divided into low‐grade (well‐ to moderately differentiated) and high‐grade based on the least or predominant differentiation analyses. We investigated the correlations between LNM and these two grading analyses.

**Results:**

LNM was present in 9.8% of patients. High‐grade tumors, as determined by least differentiation analysis, accounted for 17.0%, compared to 0.8% identified by predominant differentiation analysis. A significant association with LNM was noted for the least differentiation method (*p* < 0.05), while no such association was found for predominant differentiation (*p* = 0.18). In multivariate logistic regression, grading based on least differentiation was an independent predictor of LNM (*p* = 0.04, odds ratio 1.68, 95% confidence interval 1.00–2.83). Sensitivity and specificity for detecting LNM were 27.4% and 84.1% for least differentiation, and 2.1% and 99.3% for predominant differentiation, respectively.

**Conclusions:**

Tumor grading via least differentiation analysis proved to be a more reliable measure for assessing LNM risk in T1 CRC compared to grading by predominant differentiation.

## INTRODUCTION

The number of patients with submucosal invasive (T1) colorectal cancer (CRC) is expected to increase with the implementation of screening programs and the progress of endoscopic resection techniques such as endoscopic intermuscular dissection and endoscopic full‐thickness resection.^1^, ^2^, ^3^, ^4^ Lymph node metastasis (LNM) occurs in approximately 10% of T1 CRC cases,^5^, ^6^ so it is important to assess the need for additional surgical resection with lymph node dissection after the endoscopic resection of T1 CRCs based on the risk of LNM. Current Japanese guidelines include positive lymphovascular invasion, poorly differentiated adenocarcinoma (Por)/mucinous carcinoma (Muc)/signet‐ring cell carcinoma (Sig), depth of invasion ≥ 1000 μm, and tumor budding grade 2 or 3 as established risk factors for LNM.^7^ If one or more of these risk factors are present, the patient is deemed ‘high‐risk’ for LNM and surgical resection is recommended. On the other hand, the safety of the ‘low‐risk’ lesions without any risk factors has been validated by previous studies.^8^, ^9^, ^10^

**FIGURE 1:**
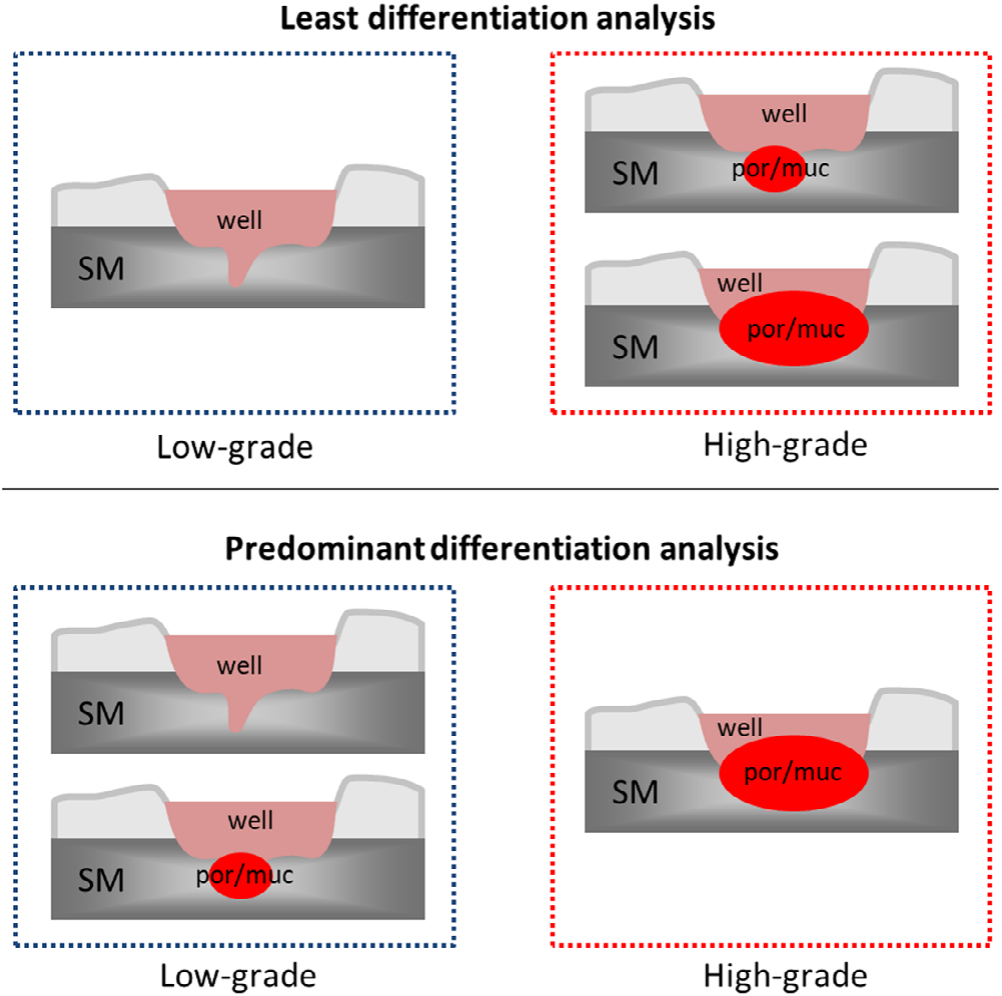
Two patterns of tumor grade classification with two or more histological differentiation components in T1 colorectal cancer. well, well‐differentiated adenocarcinoma; por, poorly differentiated adenocarcinoma; muc, mucinous carcinoma; SM, submucosal layer.

However, some issues with these guidelines need to be overcome to achieve more standardized criteria.^11^ One of these is how to assess tumor differentiation grade when the lesion consists of two or more levels of histological differentiation because the classification of tumor grade differs between guidelines.^12^ Tumor grading is classified by the least differentiation according to the World Health Organization (WHO) classification, and by the predominant differentiation using Japanese guidelines (Figure 1).^13^, ^14^ Nevertheless, even within Japan, the method of classifying tumor grade varies by institution.^8^, ^15^, ^16^, ^17^, ^18^, ^19^, ^20^, ^21^, ^22^, ^23^, ^24^, ^25^ Two recent expansive Japanese multicenter studies encompassing over 4000 T1 CRC cases diverged in their methodologies—one utilizing predominant differentiation analysis, the other least differentiation analysis (Table 1). In other words, the decision to perform additional surgical resection following endoscopic resection depends on whether the analysis is based on the least or most predominant differentiation.

**TABLE 1:** Published studies reporting variables used to assess tumor grade in Japanese institutions (the least or predominant differentiation analyses).

Author (year)	Design	Location	Least or predominant	*N*, total	Rate of high‐grade *N* (%)
Kajiwara^28^ (2023)	Multicenter‐retrospective	Japan	Predominant	4673	59 (1.3%)
Ishikawa^21^ (2022)	Single center‐retrospective	Saitama	Unclear	801	13 (1.6%)
Yamaoka^22^ (2022)	Single center‐retrospective	Shizuoka	Predominant	519	3 (0.6%)
Kudo^5^ (2021)	Multicenter‐retrospective	Japan	Least	4073	383 (9.4%)
Ouchi^16^ (2020)	Single center‐retrospective	Aichi	Predominant	458	4 (0.9%)
Mochizuki^17^ (2020)	Single center‐retrospective	Kanagawa	Least	1142	149 (13.0%)
Yasue^8^ (2019)	Single center‐retrospective	Tokyo	Least	846	93 (11.0%)
Okamura^23^ (2016)	Single center‐retrospective	Niigata	Predominant	265	7 (2.6%)
Yoshii^18^ (2014)	Single center‐retrospective	Hokkaido	Least	389	32 (8.2%)
Wada^20^ (2013)	Single center‐retrospective	Kanagawa	Predominant	120	6 (5.0%)
Nakadoi^24^ (2012)	Multicenter‐retrospective	Hiroshima	Unclear	499	19 (3.8%)
Kawaura^25^ (2007)	Single center‐retrospective	Chiba	Least	122	20 (16.4%)

This study aimed to determine which method of tumor grading classification, the least or predominant differentiation analyses, is optimal for retrospectively assessing the risk of LNM in T1 CRC.

## METHODS

### Patients and study design

We included data from all patients with pathologically diagnosed T1 CRC who underwent primary or secondary surgical resection with lymph node dissection from April 2001 to October 2021 at Showa University Northern Yokohama Hospital (Yokohama City, Japan). Patients with the following were ineligible for inclusion in this study: (1) endoscopic resection alone, (2) synchronous advanced cancer, (3) Lynch syndrome, (4) familial adenomatous polyposis, (5) inflammatory bowel disease, (6) pre‐operative chemo‐ and/or radiotherapy, and (7) missing data.

### Endpoints

The endpoint of this study was to determine whether the least or the predominant differentiation grading classification was the better variable for predicting the risk of LNM in T1 CRC when the lesion consisted of two or more levels of differentiation. In our study, tumor grading was bifurcated into low‐ and high‐grade categories. Adenocarcinomas with good differentiation (G1, characterized by >95% gland formation) and moderate differentiation (G2, with 50–95% gland formation) were designated as low‐grade. Por (G3, exhibiting <50% gland formation) were categorized as high‐grade. Furthermore, Muc and Sig were classified as high‐grade because both are considered equivalent to high‐grade due to their associated risk of LNM in Japanese guidelines (Table 2). The predominant grade was defined by the largest area among two or more components (Figure 1).

**TABLE 2:** Classification of tumor grading in this study.

High‐grade	Poorly differentiated adenocarcinoma
	Mucinous carcinoma
	Signet‐ring cell carcinoma
**Low‐grade**	**Well‐differentiated adenocarcinoma**
	Moderately differentiated adenocarcinoma
	Papillary adenocarcinoma

### Clinical and pathological data

Patient characteristics analyzed included age, sex, tumor location, tumor size, tumor morphology, lymphovascular invasion, tumor grade (the least and the predominant differentiation), tumor budding, depth of submucosal invasion, and the status of LNM. Rectum was defined as the area between the upper border of the anal canal and the lower border of the second sacral vertebra. Tumor size was measured after formalin fixation. Tumor morphology was classified as pedunculated or non‐pedunculated according to the Paris classification.^26^

All resected specimens were retrieved and immediately fixed in 10% buffered formalin. They were then cut at the point where the deepest invasion area could be exposed on the cut end surface with 2–3‐mm‐thick sections and stained with hematoxylin and eosin (H&E). All specimens were diagnosed by a single pathologist, adhering to the 2019 WHO Classification of Tumors and the prevailing guidelines of the Japanese Society for Cancer of the Colon and Rectum (JSCCR).^7^, ^14^ Lymphatic invasion was diagnosed by H&E staining and immunostaining with the D2‐40 antibody, and vascular invasion was diagnosed by double staining with H&E and Victoria Blue or Elastica van Gieson. Tumor budding was defined as a cancer cell nest consisting of one or fewer than five cells infiltrating the interstitium at the invasive margin of the cancer. On selecting the region with the most tumor budding, the front of the tumor growth was observed at 200× magnification to count the number of tumor buds: BD1, 0–4; BD2, 5–9; and BD3, ≥ 10. The depth of submucosal invasion was classified according to JSCCR guidelines as <1000 μm (T1a) and ≥ 1000 μm (T1b). Criteria for subsequent bowel resection were applied when any of the following parameters were identified in the endoscopically resected specimen, in accordance with the JSCCR guidelines: (1) T1b (depth of submucosal invasion ≥1000 μm), (2) positive lymphovascular invasion, (3) poorly differentiated adenocarcinoma, signet‐ring cell carcinoma, or mucinous carcinoma, or (4) a budding grade of BD2/3 at the point of deepest invasion. In our study, we utilized the least differentiation analysis to assess tumor grading for the criteria guiding secondary surgery. Operative specimens were used as the gold standard for the presence or absence of LNM.

### Statistical analysis

Continuous variables were reported as the mean ± standard deviation. Dichotomous variables were compared using chi‐squared or Fisher's exact tests, as appropriate. Multivariate logistic regression analysis regarding LNM was subsequently performed to calculate odds ratios (ORs) and 95% confidence intervals (CIs). McNemar's test was used to compare the sensitivity and specificity between the two methods. All statistical analyses were performed using R for Windows 4.0.3. All *p*‐values were two‐sided, and *p* < 0.05 was considered statistically significant.

### Ethical considerations

This study was approved by the institutional review board of Showa University Northern Yokohama Hospital (approval no. 20H022) and was registered with the University Hospital Medical Network Clinical Trials Registry (UMIN 000042622). Written informed consent was obtained from all patients before treatment.

## RESULTS

### Study cohort

**FIGURE 2:**
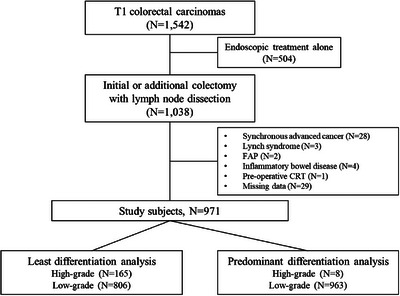
Study flow chart.

Figure 2 shows the study flowchart. A total of 1038 patients with pT1 CRC underwent initial or additional surgical resection with lymph node dissection during the study period. Of these, 28 patients with synchronous invasive cancers at the time of resection, three with suspected Lynch syndrome, two with familial adenomatous polyposis, four with inflammaroty bowel disease, one with pre‐operative chemo‐ and/or radiotherapy, and 29 with missing data from patient files and local registries for variables were excluded. Thus, 971 patients were eligible and included in the analyses. Table 3 presents the clinicopathological characteristics of the patients in this study. The LNM rate was 9.8% (95/971), and the mean number of dissected lymph nodes was 20 ± 11 (median, 19).

**TABLE 3:** Patient baseline characteristics (*n* = 971).

	Total (*n* = 971)	LNM‐positive (*n* = 95)	LNM‐negative (*n* = 876)	*p*‐value
Age, mean years ± SD	66 ± 12	64 ± 11	66 ± 11	0.02
Sex, *n* (%)				0.02
Male	599 (61.7)	48 (50.5)	551 (62.9)	
Female	372 (38.3)	47 (49.5)	325 (37.1)	
Tumor location, *n* (%)				0.58
Colon	695 (71.6)	76 (80.0)	720 (82.2)	
Rectum	276 (28.4)	19 (20.0)	156 (17.8)	
Tumor size, mean mm ± SD	21 ± 15	21 ± 13	21 ± 12	0.93
Tumor morphology, *n* (%)				1.00
Pedunculated	94 (9.7)	9 (9.5)	85 (9.7)	
Non‐pedunculated	877 (90.3)	86 (90.5)	791 (90.3)	
Initial treatment				0.45
Endoscopic resection	431 (44.4)	46 (48.4)	385 (43.9)	
Surgical resection	540 (55.6)	49 (51.6)	491 (56.1)	
Lymphovascular invasion, *n* (%)				<0.0001
Positive	536 (55.2)	88 (92.6)	448 (51.1)	
Negative	435 (44.8)	7 (7.4)	428 (48.9)	
Histological differentiation (least), *n* (%)				<0.01
High‐grade (Por/Muc/Sig)	165 (17.0)	26 (27.4)	139 (15.9)	
Low‐grade (Well/Mod/Pap)	806 (83.0)	69 (72.6)	737 (84.1)	
Histological differentiation (predominant), *n* (%)				0.18
High‐grade (Por/Muc/Sig)	8 (0.8)	2 (2.1)	6 (0.7)	
Low‐grade (Well/Mod/Pap)	963 (99.2)	93 (97.9)	870 (99.3)	
Tumor budding, *n* (%)				<0.0001
Grade 1	736 (75.8)	54 (56.8)	682 (77.9)	
Grade 2/3	235 (24.2)	41 (43.2)	194 (22.1)	
Depth of submucosal invasion, *n* (%)				0.73
pT1a	110 (11.3)	9 (9.5)	101 (11.5)	
pT1b	861 (88.7)	86 (90.5)	775 (88.5)	

Results are expressed as number of patients (%).

Abbreviations: LNM, lymph node metastasis, Mod, moderately differentiated adenocarcinoma; Muc, mucinous carcinoma; Pap, papillary adenocarcinoma; Por, poorly differentiated adenocarcinoma; SD, standard deviation; Sig, signet‐ring cell carcinoma; Well, well‐differentiated adenocarcinoma.

### The least grade versus predominant differentiation grading classification

Table 4 compares the predictive ability of the least and predominant differentiation analysis for the presence of LNM as a single predictor. The rate of the high grade was 17.0% (95% CI, 14.7%–19.5%) in the least differentiation analysis and 0.8% (95% CI, 0.45%–1.6%) in the predominant differentiation analysis. The sensitivity for LNM was 27.4% (95% CI, 18.7%–37.5%) in the least differentiation analysis and 2.1% (95% CI, 0.3%–7.4%) in the predominant differentiation analysis (*p* < 0.01), while the specificity was 84.1% (95% CI, 81.5%–86.5%) and 99.3% (98.5%–99.7%), respectively (*p* < 0.01).

**TABLE 4:** Predictive value of tumor grade for lymph node metastasis as a single predictor (the least vs. predominant differentiation analyses).

	Least % (95% CI)	Predominant % (95% CI)	*p*‐value
Rates of high‐grade	165/971 17.0% (14.7%–19.5%)	8/971 0.8% (0.4%–1.6%)	<0.01
Sensitivity	26/95 27.4% (18.7%–37.5%)	2/95 2.1% (0.3%–7.4%)	<0.01^*^
Specificity	737/876 84.1% (81.5%–86.5%)	870/876 99.3% (98.5%–99.7%)	<0.01^*^

* McNemar's test, CI, confidence interval.

The association between various factors and LNM, as well as the OR for clinicopathological determinants in both univariate and multivariate logistic regression analyses, are delineated in Tables 5 and 6. Univariate analysis revealed a significant correlation between LNM and tumor grade with the least differentiation analysis (*p* < 0.01), in contrast to the non‐significant association with tumor grade via predominant differentiation analysis (*p* = 0.18). Multivariate logistic regression substantiated tumor grade via least differentiation analysis as an independent prognostic factor (*p* = 0.04, OR 1.68, 95% CI 1.00–2.83), whereas tumor grade based on predominant differentiation did not retain independent predictive value (*p* = 0.40, OR 2.07, 95% CI 0.38–11.2).

**TABLE 5:** Relationships between clinicopathological factors and least differentiation analysis.

	Univariate analysis			Multivariate analysis	
	LNM+ (*n* = 95)	LNM− (*n* = 876)	*p*‐value	OR (95% CI)	*p*‐value
Age (<70 years)	56 (58.9)	504 (57.5)	0.83	0.96 (0.61–1.51)	0.85
Sex (male)	48 (50.5)	551 (62.9)	0.02	0.52 (0.33–0.81)	<0.01
Tumor location (colon)	76 (80.0)	720 (82.2)	0.58	1.03 (0.58–1.83)	0.90
Tumor size (≥20 mm)	44 (46.3)	409 (46.7)	0.99	1.06 (0.67–1.67)	0.80
Tumor morphology (pedunculated)	9 (9.5)	85 (9.7)	1.00	1.13 (0.50–2.55)	0.76
Initial treatment (Surgical resection)	49 (51.6)	491 (56.1)	0.45	0.83 (0.53–1.32)	0.43
Lymphovascular invasion (+)	88 (92.6)	448 (51.1)	<0.0001	11.47 (5.15–25.5)	<0.0001
Histological differentiation (least) (Por/Muc/Sig)	26 (27.4)	139 (15.9)	<0.01	1.68 (1.00–2.83)	0.04
Tumor budding (BD 2 or 3)	41 (43.2)	194 (22.1)	<0.0001	1.53 (0.96–2.43)	0.07
Depth of submucosal invasion (T1b)	86 (90.5)	775 (88.5)	0.73	0.82 (0.37–1.80)	0.62

Results are expressed as number of patients (%).

Abbreviations: CI, confidence interval; LNM, lymph node metastasis; Muc, mucinous carcinoma; OR, odds ratio; Por, poorly differentiated adenocarcinoma; Sig, signet‐ring cell carcinoma.

**TABLE 6:** Relationships between clinicopathological factors and lymph node metastasis (Predominant differentiation analysis).

	Univariate analysis			Multivariate analysis	
	LNM+ (*n* = 95)	LNM− (*n* = 876)	*p*‐value	OR (95% CI)	*p*‐value
Age (<70 years)	56 (58.9)	504 (57.5)	0.83	0.96 (0.61–1.51)	0.89
Sex (male)	48 (50.5)	551 (62.9)	0.02	0.52 (0.33–0.81)	<0.01
Tumor location (colon)	76 (80.0)	720 (82.2)	0.58	1.03 (0.58–1.83)	0.88
Tumor size (≥20 mm)	44 (46.3)	409 (46.7)	0.99	1.06 (0.67–1.67)	0.79
Tumor morphology (pedunculated)	9 (9.5)	85 (9.7)	1.00	1.13 (0.50–2.55)	0.87
Initial treatment (Surgical resection)	49 (51.6)	491 (56.1)	0.45	0.83 (0.53–1.32)	0.39
Lymphovascular invasion (+)	88 (92.6)	448 (51.1)	<0.0001	11.47 (5.15–25.5)	<0.0001
Histological differentiation (Predominant) (Por/Muc/Sig)	2 (2.1)	6 (0.7)	0.18	2.07 (0.38–11.2)	0.40
Tumor budding (BD 2 or 3)	41 (43.2)	194 (22.1)	<0.0001	1.53 (0.96–2.43)	0.04
Depth of submucosal invasion (T1b)	86 (90.5)	775 (88.5)	0.73	0.82 (0.37–1.80)	0.66

Results are expressed as number of patients (%).

Abbreviations: CI, confidence interval; LNM, lymph node metastasis; Muc, mucinous carcinoma; OR, odds ratio; Por, poorly differentiated adenocarcinoma; Sig, signet‐ring cell carcinoma.

## DISCUSSION

In this study, we investigated which method of assessing histological differentiation grade as a risk factor for LNM in patients with T1 CRC (the least or predominant differentiation analyses) was superior with respect to diagnostic performance. We concluded that tumor grade, when assessed through least differentiation analysis, emerged as an independent prognostic factor for LNM, unlike when evaluated via predominant differentiation. In addition, the least differentiation analysis showed higher sensitivity, reducing the risk of misclassifying patients with LNM as negative in T1 CRC, although the predominant differentiation analysis showed higher specificity, preventing potentially unnecessary surgeries.

The WHO classification uses the least differentiation analysis, while JSCCR guidelines adopt the predominant differentiation analysis. However, the assessment of tumor grade according to the least or predominant component actually depends on the institution in Japan (Table 1). Among the nine single institutions, the least differentiation analysis was used in four, the predominant differentiation analysis was adopted in four, and the method used in the remaining one institution was unclear.^8^, ^16^, ^17^, ^18^, ^20^, ^21^, ^22^, ^23^, ^25^ When classified by the predominant differentiation analysis, 0.8% (8/971) of the cohort was defined as high‐grade in the present study which is equal to the 0.6%–5.0% found in the previous studies with the predominant differentiation analysis.^16^, ^20^, ^22^, ^23^ It suggests that this method may lack the sensitivity to act as a risk factor. This study underscores and contributes to the discourse on this issue. When classified by the least differentiation analysis, 17.0% (165/971) of the cohort was defined as high‐risk for LNM in the present study compared with 8.2%–16.4% in previous studies.^8^, ^17^, ^18^, ^25^

Sensitivity was higher when using the least differentiation analysis (27.4%) compared with the predominant differentiation analysis (2.1%). This suggests that classifying tumor grade according to the least differentiation analysis is preferable to avoid missing an LNM‐positive case. However, specificity was lower using the least differentiation analysis (84.1%) than the predominant differentiation analysis (99.3%), which could lead to an increase in unnecessary surgeries. Our dataset lacked LNM‐positive cases that were deemed high risk through least differentiation analysis yet low risk by predominant differentiation analysis, thus precluding a direct assessment of least differentiation's efficacy. This could be attributable to the high incidence of T1b (88.7%) and the strong correlation between lymphovascular invasion and LNM. In this study, the assessment of lymphovascular invasion, one of the most reliable predictors for LNM, was supplemented with immunohistochemical staining techniques, including D2‐40 for lymphatic invasion and Victoria Blue/Elastica van Gieson for vascular invasion, in addition to standard techniques. As a result, the positivity rate of lymphovascular invasion (55.2%) was higher than that reported in other studies (39%–42%), leading to a higher odds ratio for LNM of 11.5, compared to 4.2–8.1 reported in previous studies.^8^, ^27^

This study had several limitations. First, it was a retrospective single‐center study limited by a relatively small sample size compared with recent large‐scale multicenter studies of over 4000 T1 CRC cases.^5^, ^28^ Secondly, our analysis did not consider T1 CRCs managed solely through endoscopic treatment, where LNM status remained undetermined. Consequently, this exclusion encompassed 29 high‐grade cases identified by least differentiation analysis and none by predominant differentiation analysis, potentially introducing selection bias into our study results.

In conclusion, tumor grading with the least differentiation analysis was an independent risk factor for LNM in T1 CRC. The least differentiation analysis of assessing tumor grading had a higher sensitivity for LNM in T1 CRC than the predominant differentiation analysis.

## CONFLICT OF INTEREST STATEMENT

The authors declare no conflict of interest.
